# The relationship between negative workplace gossip and thriving at work among Chinese kindergarten teachers: the roles of psychological contract breach and *bianzhi*

**DOI:** 10.3389/fpsyg.2023.1198316

**Published:** 2023-07-19

**Authors:** Can He, Tongtong Feng, Jie Xiong, Hua Wei

**Affiliations:** ^1^College of Education Science, Hubei Normal University, Huangshi, China; ^2^Human Resources Office, Hubei University of Arts and Science, Xiangyang, China; ^3^Center for Mental Health, Wuhan University of Technology, Wuhan, Hubei, China; ^4^Normal College, Qingdao University, Qingdao, Shandong, China

**Keywords:** negative workplace gossip, thriving at work, psychological contract breach, *bianzhi*, kindergarten teachers

## Abstract

**Introduction:**

Exploring the influencing factors and functioning mechanisms of thriving at work is of practical significance both for teachers and kindergartens. Based on the socially embedded model of thriving at work, this study aimed to examine the association between negative workplace gossip and thriving at work. The mediating role of psychological contract breach and the moderating role of *bianzhi* were also examined.

**Methods:**

A total of 1105 Chinese kindergarten teachers were chosen to complete a questionnaire on negative workplace gossip, psychological contract breach, and thriving at work.

**Results:**

The results demonstrated that negative workplace gossip was positively associated with psychological contract breach and negatively associated with thriving at work. In addition, psychological contract breach was negatively associated with thriving at work. According to the mediation model test, psychological contract breach was a mediating factor between negative workplace gossip and thriving at work. The impact of psychological contract breach on thriving at work could be further moderated by *bianzhi*.

**Conclusion:**

This study complements knowledge systems about the influential factors and functional mechanisms of thriving at work. In practical terms, this study offers a fresh and innovative perspective for kindergartens seeking to enhance teachers’ thriving at work.

## Introduction

Kindergarten teachers face a high level of pressure due to the complex nature of their work and the specificity of their educational objects, which makes them one of the most stressed occupational groups (Moriarty et al., 2001). In China, kindergarten teachers face even more significant work pressure and workload due to the severe shortage of teachers and large class sizes (Hong et al., 2021). These challenges lead to the loss of work vitality and enthusiasm, resulting in job burnout and health-related concerns (Huang et al., 2017; Wang et al., 2017; Yang and Lin, 2018). As early childhood education (ECE) is a crucial foundation stage for school education, and the quality of ECE significantly affects the development of young children and their subsequent learning and growth. Therefore, kindergarten teachers play an essential role in ECE as the direct implementers of school education. Improving work vitality and physical and mental health of kindergarten teachers is imperative for the development of ECE and the ECE workforce in China. As a signal of individual progress and growth, thriving at work helps employees maintain high energy, reduce burnout, and enhance physical and mental health (Spreitzer et al., 2005; Porath et al., 2012; Walumbwa et al., 2018). Thriving at work refers to the psychological state in which employees feel both a sense of vitality and learning (Spreitzer et al., 2005; Porath et al., 2012). As two essential components of thriving at work, vitality indicates the positive feeling of having available energy, and learning is the state of obtaining and using knowledge and abilities. Beyond improving performance and reducing turnover rates, thriving at work can positively affect employees in terms of career development initiatives and overall health (Porath et al., 2012; Walumbwa et al., 2018). This is especially crucial for kindergarten teachers, a profession demanding constant vitality and passion. These educators serve young children who are in an exploratory developmental phase, marked by curiosity and endless energy, yet their cognitive and emotional regulation skills are still maturing. This context highlights the necessity for teachers to maintain high levels of professional enthusiasm and vigor, enabling them to effectively cater to the needs of young children. Therefore, examining the influencing factors and functioning mechanisms of thriving at work is of practical significance for kindergarten teachers and organizations.

Previous studies have shown that various deviant workplace behaviors, such as workplace incivility and workplace ostracism, can negatively impact thriving at work (Kleine et al., 2019; Abid and Contreras, 2022; Zhang et al., 2023). However, despite being a highly prevalent form of interpersonal deviance in the workplace, negative workplace gossip has received relatively little research attention. Negative workplace gossip refers to the informal and unfavorable discussions or the dissemination of rumors about a third member of the organization who is not present (Chandra and Robinson, 2009; Cheng et al., 2020). It is pervasive and unavoidable in workplace settings. Negative workplace gossip has been found to have a range of negative impacts on the gossiped targets, such as increased engagement in political acts (Cheng et al., 2020), emotional exhaustion (Wu et al., 2018), reduced service performance (Ye et al., 2019), and even the intention to resign (He and Wei, 2022). In the context of China, a collectivist nation that places significant importance on relationships, interpersonal relationships carry more weight in Chinese society than in Western society. Thus, negative workplace gossip, as a form of negative interpersonal communication, could be detrimental to the thriving at work Chinese kindergarten teachers.

### Negative workplace gossip and thriving at work

The socially embedded model of thriving at work (SEMT) suggests that employees are more likely to thrive in special work contexts, and that increases in supportive contextual features (e.g., an atmosphere of respect and trust, freedom to make decisions) are necessary for thriving at work (Spreitzer et al., 2005; Kleine et al., 2019). When individuals are situated in a climate of respect, they are likely to engage in agentic work behaviors, which in turn contributes to thriving at work. According to the SEMT, the climate of respect is an important contextual feature. However, the low-quality interpersonal treatment in negative workplace gossip (e.g., verbal abuse, and personal insults) can create an organizational climate of disrespect. This climate of disrespect can reduce agentic work behaviors, weaken vitality and learning experience, and impair thriving at work. Previous studies have demonstrated that negative workplace gossip undermines thriving at work (Qu et al., 2022; Zhu et al., 2022). Accordingly, we propose H1: Negative workplace gossip is negatively correlated with thriving at work.

### The mediation role of psychological contract breach

According to the SEMT, contextual features of organizations, such as a climate of respect, are important factors affecting thriving at work, which include both objective and subjective perceived features. Negative workplace gossip is an objective contextual feature that reflects organizational members’ disrespect for individuals. In addition to objective contextual features, subjective perceived contextual features also affect thriving at work (Yan et al., 2022). We suggest that psychological contract breach is likely to be a significant subjective perceived contextual feature that impacts thriving at work. Psychological contract breach refers to a perception of employees that the organization has not fulfilled the obligations in psychological contracts (Morrison and Robinson, 1997). Unlike negative workplace gossip, psychological contract breach is individuals’ subjective perception of disrespect by the organization. A previous study has confirmed that subjectively perceived features, such as psychological contract breach, can impair thriving at work (Qaiser et al., 2020). After psychological contract breach occurs, individuals may need to expend energy to cope with the negative emotions triggered by the breach, making it difficult to focus on work tasks and explore new work behaviors, ultimately reducing agentic work behaviors, and undermining the individual’s vitality and learning experience at work.

Furthermore, individuals’ subjective perceptions of contextual features can be influenced by objective contextual features. Employees generally expect organizations to create a safe and respectful work environment and treat them with respect and dignity (Salin and Notelaers, 2017). Negative workplace gossip can violate employees’ expectations that they should be treated with respect in the organization, leading to the perception that the organization is not meeting its commitments and resulting in psychological contract breach. Previous studies have also confirmed that workplace deviant behaviors can cause the breach of psychological contracts (Bari et al., 2022; Chaudhary and Islam, 2022). Based on the aforementioned studies, we propose H2: Psychological contract breach may mediate the relationship between negative workplace gossip and thriving at work.

### The moderation role of *bianzhi*

Although negative workplace gossip may affect thriving at work through psychological contract breach, this effect could be mitigated by other variables. Based on relevant theories and prior research (He and Wei, 2022; Wang et al., 2022), the association between psychological contract breach and thriving at work may be moderated by *bianzhi*. In the Chinese education system, the term “*bianzhi*” refers to a faculty position formally recognized and appointed by the local department of education, which is similar to tenure in the American educational system (Hu et al., 2015, 2016). *Bianzhi* is particularly significant to teachers in China since it represents their professional identity. The benefits of *bianzhi* include lifetime employment, public housing subsidies, reliable health insurance, and stable salaries. Although teachers with and without *bianzhi* may have the same responsibilities, they differ significantly in employment stability, social status, salary, and subsidies (He and Wei, 2022). Compared with those without *bianzhi*, teachers with *bianzhi* are more vulnerable to the psychological contract breach. Prior research suggested that *bianzhi* can promote kindergarten teachers’ organizational identification (Liu et al., 2021). However, the effects of organizational identification are not always positive (Conroy et al., 2017). Highly identified employees usually have higher expectations of the organization and its members. If the expectations are violated, they are more likely to experience disappointment (Conroy et al., 2017). As for teachers with *bianzhi*, when the organization breaks its commitments or obligations, they may experience a greater degree of psychological contract breach and violation, thus exacerbating the negative impact on thriving at work. On the contrary, teachers without *bianzhi* have no excessive expectations of the organization. They might not react strongly when a psychological contract is broken.

In addition, the types of psychological contracts may differ between teachers with *bianzhi* and those without it. Generally, the content of these psychological contracts can be categorized as either transactional or relational. Transactional contracts are characterized by short-term, specific, and monetizable obligations requiring limited involvement from the parties, whereas relational contracts entail long-term, extensive, open-ended obligations that incorporate socio-emotional components like loyalty and support (Rousseau and Parks, 1993). Teachers with *bianzhi*, who enjoy secure lifelong employment and stable salaries, may lean more toward relational psychological contracts, emphasizing long-term, implicit, social exchanges, such as career development, promotion opportunities, and a supportive work environment. Conversely, teachers without *bianzhi* be more inclined toward transactional psychological contracts, focusing on short-term, explicit, economic exchanges, such as remuneration and work tasks. Prior studies have indicated that the type of contract significantly moderates the relationship between psychological contract breach and personal and organizational outcomes (Raja et al., 2011; Chiang et al., 2012; Jamil et al., 2013). Psychological contract breach tends to provoke stronger reactions from employees with relational contracts than those with transactional ones. When they perceive a breach, employees with relational contracts are more likely to exhibit reduced performance, decreased satisfaction, and increased intentions to quit (Zhao et al., 2007; Raja et al., 2011). Therefore, teachers with *bianzhi* may react more intensely to psychological contract breach, potentially leading to a more profound negative effect on their thriving at work. Based on the above studies, we propose H3: B*ianzhi* acts as a moderator in the relationship between psychological contract breach and thriving at work. Specifically, the negative relationship between psychological contract breach and thriving at work is stronger for teachers with *bianzhi* than for those without it.

### The present study

**Figure 1:**
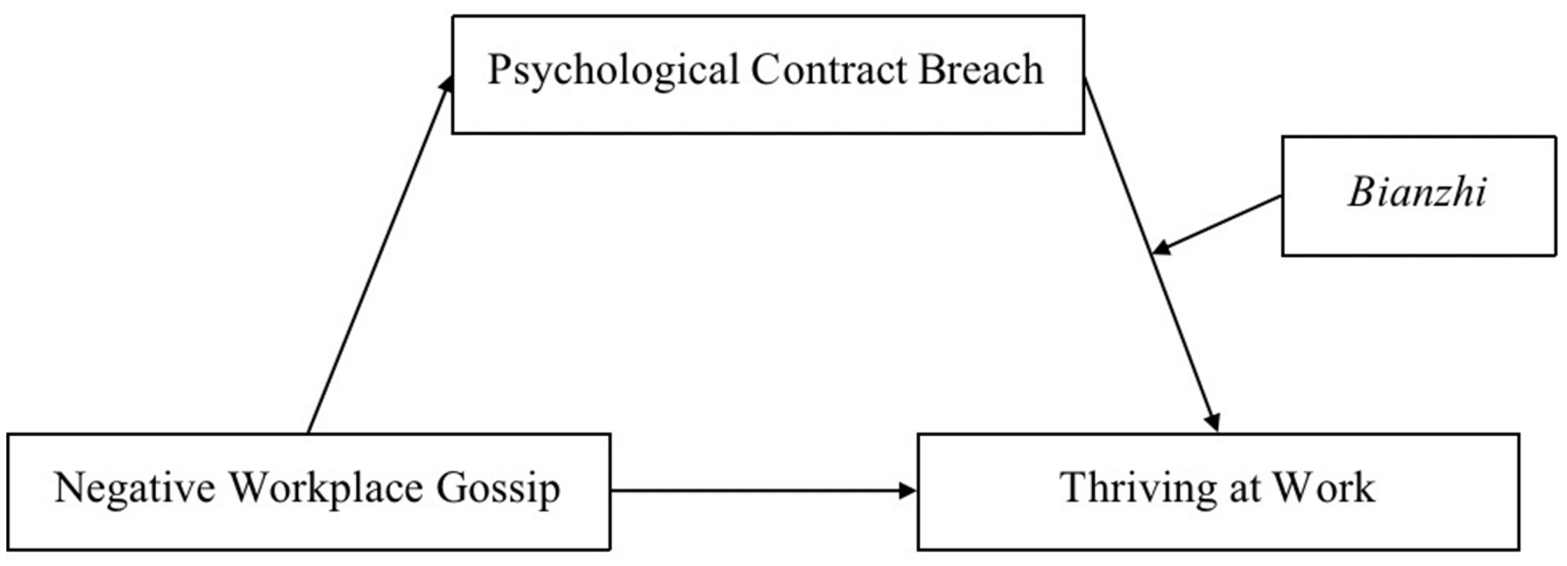
The hypothetical model.

This study aims to further investigate how negative workplace gossip affects thriving at work among kindergarten teachers. First, we intend to examine the link between negative workplace gossip and thriving at work. Second, we intend to investigate the mediating function of psychological contract breach and the moderating function of *bianzhi* to reveal the functioning mechanisms. The results of this study can be used to provide valuable insights for kindergartens to enhance the thriving at work of teachers. Figure 1 illustrates the hypothetical model.

## Materials and methods

### Participants and procedure

This study was approved by the Ethics Committee for Scientific Research of our institution. We adopted convenience sampling to recruit participants through an online questionnaire platform called Questionnaire Star. In total, 1,105 kindergarten teachers from Hubei Province participated in this investigation. Specifically, the investigation was performed in October 2022 with the assistance of the Huangshi Municipal Bureau of Education and distributed to kindergarten teachers through their respective principals. The survey was carried out in the Mandarin Chinese language. Before the investigation, we obtained informed consent from the participants. The investigation was completed independently, anonymously, and honestly by each participant. Among them, 292 (26.4%) had *bianzhi*, and 813 (73.6%) did not have *bianzhi* (median age = 31.45, SD = 7.09). In addition, they were all female and full-time teachers.

### Measures

#### Negative workplace gossip

The Chinese version of a three-item scale proposed by Chandra and Robinson (2009) was utilized to assess negative workplace gossip (see Appendix). The items were graded on a five-point scale ranging from 1 = never to 5 = daily, with higher scores indicating a higher degree of negative gossip. The reliability and validity of the scale has been demonstrated in the previous study (Xing et al., 2021). In the current study, Cronbach’s alpha was 0.80.

#### Psychological contract breach

The Chinese version of the psychological contract breach scale developed by Robinson and Morrison (2000) was employed (see Appendix). The scale consisted of five items, each rated on a five-point scale ranging from 1 (completely disagree) to 5 (completely agree), with some questions being reverse-scored. Previous research has confirmed that the scale is suitable for Chinese samples (Zhang et al., 2017). In the current study, Cronbach’s alpha was 0.84.

#### Thriving at work

Thriving at work was measured by the Chinese version of a ten-item scale developed by Porath et al. (2012) (see Appendix). The scale has demonstrated good psychometric proprieties in the Chinese cultural context (Zeng et al., 2020). Participants answered questions based on a seven-point scale ranging from 0 (not agree at all) to 7 (totally agree), with some questions being reverse-scored. In the current study, Cronbach’s alpha for the scale was 0.84.

#### Data analyses

In the present study, SPSS 25.0 and Mplus 8.3 were used for data analysis. We employed the structural equation model to test common method variance, followed by descriptive statistics for all variables. Afterward, we conducted the correlation analysis to investigate the associations between each variable. Finally, we adopted SPSS macro program PROCESS to test the moderated mediating model (Hayes, 2018). The mediation effect was tested by model 4 in the PROCESS template, and the moderated mediation effect was tested by model 14 in the template. The z-scores for continuous variables were produced before regression analysis because PROCESS Marco provided no standardized regression coefficients. Furthermore, we generated a dummy variable to represent *bianzhi*, where teachers with *bianzhi* were coded as 1 and those without *bianzhi* were coded as 0. The confidence interval (CI) based on the indirect effects of the original sample was approximated using 5,000 bootstrap samples created during the processing of the PROCESS Macro, and no zero 95% CI suggests statistical significance. Age was also controlled in each regression model.

## Results

### Common method deviation test

We performed confirmatory factor analysis to examine the hypothesis that a single factor could explain all the variance of the study data (Podsakoff et al., 2003). The test was conducted with Mplus 8.3, and the results demonstrated a poor model fit (*X*^2^/df = 61.85, RMSEA = 0.24, CFI = 0.41, and TLI = 0.33), proving the absence of common method bias.

### Preliminary analyses

The descriptive results for all variables are presented in Table 1. It can be seen that negative workplace gossip was negatively related with thriving at work (*r* = −0.23, *p* < 0.01) and positively related with psychological contract breach (*r* = 0.31, *p* < 0.01). Psychological contract breach was negatively related with thriving at work (*r* = −0.34, *p* < 0.01).

**Table 1:** Means, standard deviations, and correlation coefficients.

Variable	*M*	*SD*	1	2	3	4
1. NWG	1.51	0.76	—			
2. PVB	2.48	0.66	0.31^**^	—		
3. *Bianzhi*	0.26	0.44	0.08^*^	−0.10^**^	—	
4. TW	4.11	0.55	−0.23^**^	−0.34^**^	−0.03	—

*N* = 1105.

NWG, negative workplace gossip; PCB, psychological contract breach; TW, thriving at work.

^*^p < 0.05, ^**^p < 0.01.

### Testing for the mediating role of psychological contract breach

We employed Model 4 in the SPSS macro-PROCESS to investigate the potential relationship between negative workplace gossip and thriving at work and the possible mediating function of psychological contract breach. Table 2 shows the results of the analysis. The findings show that negative workplace gossip was negatively associated with thriving at work (*β* = −0.23, *p* < 0.01) and positively associated with psychological contract breach (*β* = 0.31, *p* < 0.01). When psychological contract breach was added as a mediator into the regression model, negative workplace gossip was negatively associated with thriving at work (*β* = −0.13，*p* < 0.01), and psychological contract breach was negatively associated with thriving at work (*β* = −0.30，*p* < 0.01). As demonstrated by the bias-corrected bootstrapping mediation test, psychological contract breach mediated the relationship between negative workplace gossip and thriving at work, supporting H1 and H2. Specifically, the mediating effect was-0.09, Boot *SE* = 0.01, and 95% CI = [−0.12，-0.07]. Moreover, the mediation effect was responsible for 39.13% of the total effect.

**Table 2:** Mediation analyses.

	Model (1) (Criterion = TW)			Model (2) (Criterion = PCB)			Model (3) (Criterion = TW)		
	*β*	*SE*	*t*	*β*	*SE*	*t*	*β*	*SE*	*t*
Age	0.01	0.00	2.24^*^	0.01	0.00	3.21^**^	0.01	0.00	3.33^**^
NWG	−0.23	0.03	−7.81^**^	0.31	0.03	10.91^**^	−0.13	0.03	−4.55^**^
PCB							−0.30	0.03	−10.28^**^
*R*^2^	0.06			0.10			0.14		
*F*	34.18^**^			62.79^**^			60.18^**^		

NWG, negative workplace gossip; PCB, psychological contract breach; TW, thriving at work. ^*^p < 0.05, ^**^p < 0.01.

### Testing for moderated mediation

**Figure 2:**
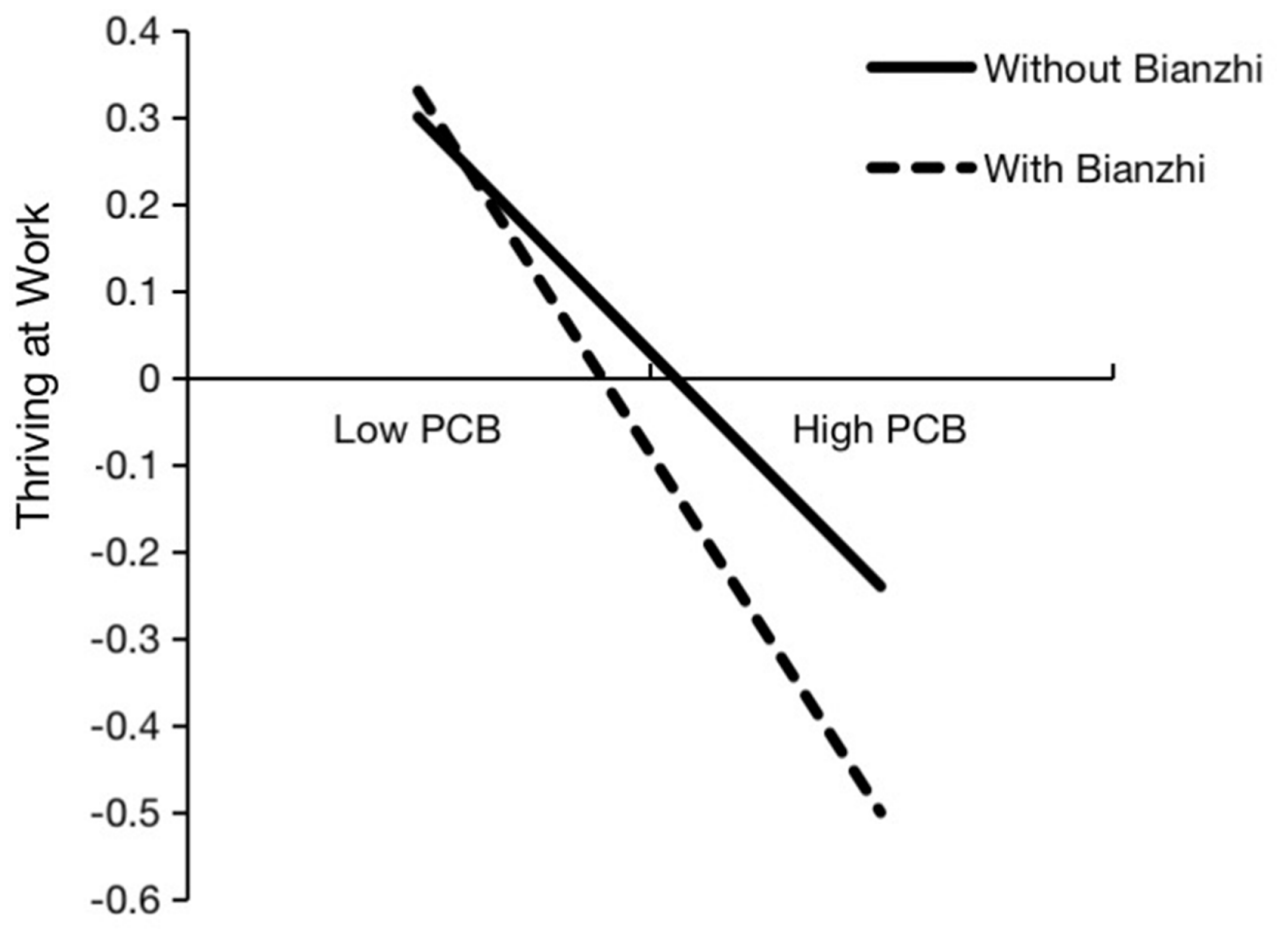
Interaction of psychological contract breach and *bianzhi* on thriving at work.

We adopted model 14 in the SPSS macro-PROCESS to determine whether *bianzhi* mitigated the correlation between psychological contract breach and thriving at work. Table 3 presents the results of the analysis. The regression model indicated that the interaction between psychological contract breach and *bianzhi* was correlated with thriving at work (*β* = −0.15, *p* < 0.05). Simple slope tests revealed that for teachers without *bianzhi*, psychological contract breach was negatively correlated with thriving at work (*B*simple = −0.27, *t* = −7.98, *p* < 0.01), while for teachers with *bianzhi*, this correlation remained significant but stronger (*B*simple = −0.41, *t* = −7.48, *p* < 0.01). The interaction is plotted in Figure 2. These results suggest that *bianzhi* moderates the relationship between psychological contract breach and thriving at work, supporting H3.

**Table 3:** Moderated mediation analyses.

	Model (1) (Criterion = PCB)			Model(2) (Criterion = TW)		
	*β*	*SE*	*t*	*β*	*SE*	*t*
Age	0.01	0.00	3.21^**^	0.01	0.00	3.43^**^
NWG	0.31	0.03	10.91^**^	−0.13	0.03	−4.28^**^
PCB				−0.27	0.03	−7.98^**^
*Bianzhi*				−0.11	0.06	−1.67
PCB × *Bianzhi*				−0.15	0.06	−2.30^*^
*R*^2^	0.10			0.15		
*F*	62.79^**^			37.75^**^		

NWG, negative workplace gossip; PCB, psychological contract breach; TW, thriving at work. ^*^p < 0.05, ^**^p < 0.01.

## Discussion

Based on the SEMT, the correlation between negative workplace gossip and thriving at work among Chinese kindergarten teachers was explored. The mediating function of psychological contract breach and the moderating function of *bianzhi* were also investigated. The results revealed a negative correlation between negative workplace gossip and thriving at work. Negative workplace gossip can create a disrespectful atmosphere, deplete psychological resources, and make it difficult for individuals to feel energized about their work. It can also damage reputations and cause individuals’ gradual exclusion from the group, limiting their access to learning and development opportunities, and ultimately undermining thriving at work. It is noteworthy that negative workplace gossip affects thriving at work through the mediating effect of psychological contract breach. In other words, higher levels of psychological contract breach were associated with lower levels of thriving at work. Additionally, the correlation between psychological contract breach and thriving at work was moderated by *bianzhi*. Specifically, for kindergarten teachers with *bianzhi* compared to those without *bianzhi*, psychological contract breach had a larger effect on thriving at work.

### Theoretical contributions

Several theoretical implications can be concluded from the findings of this study. First, by focusing on thriving at work, a less-studied dependent variable, this study further explores workplace gossip. Previous studies on workplace gossip have mainly focused on employee attitudinal and behavioral outcomes such as turnover intention, organizational citizenship behavior, and job performance (Kong, 2018; Xie et al., 2019; Ye et al., 2019; Xie et al., 2020; He and Wei, 2022). This study contributes to the existing literature by exploring the impact of negative workplace gossip on the positive psychological states of employees. To the best of our knowledge, this study first investigates the relationship between negative workplace gossip and thriving at work in kindergarten settings. By shifting the research focus from the finance and service industries (Qu et al., 2022; Zhu et al., 2022) to early childhood education, this study further expands the research scope. Unlike profit-centric economic organizations such as finance and service industries, kindergarten organizations place a more substantial emphasis on their staff’s dedication. Particularly in a traditionally collectivist society like China, both the governmental education departments and the general public place immense importance on the moral conduct of kindergarten teachers and stress the creation of a harmonious and caring kindergarten environment. Consequently, kindergarten teachers may have higher expectations for a just and harmonious work environment and cordial colleague relationships. This could potentially amplify the negative impact of negative workplace gossip on their thriving at work. Although this study was conducted in the Chinese context, the phenomenon it investigates is prevalent in kindergarten workplace settings (Cantisano et al., 2007; Royer and Moreau, 2016; Çetin et al., 2020). Therefore, the findings of this study have implications for research on kindergarten teachers in different cultural contexts. However, to further validate the generalization of the results, more studies need to be conducted in different regions. Additionally, it is worth noting that in a collectivist cultural context, which emphasizes interpersonal relationships, the negative impact of workplace gossip on kindergarten teachers’ work thriving could potentially be more significant. Therefore, future research could further explore and compare the relationship between negative workplace gossip and thriving at work among kindergarten teachers within different cultural contexts, to enhance our understanding and knowledge of this issue.

Second, this study complements the limited literature that examines psychological contract breach as a mediating factor in the connection between negative workplace gossip and employee work-related outcomes. Although psychological contract breach has been well studied as a mediator between workplace contexts and work-related outcomes (Kayani and Alasan, 2021; Bari et al., 2022; Chaudhary and Islam, 2022), its influence in the negative workplace gossip phenomenon has not been explored. This study expands research on the mechanisms underlying the dysfunctional outcomes of negative workplace gossip by drawing on the SEMT (Kong, 2018; Ye et al., 2019; Xie et al., 2020; He and Wei, 2022). Specifically, this study confirms for the first time the mediating role of psychological contract breach in the outcomes of workplace interpersonal deviance (Bari et al., 2022). Furthermore, this study broadens the applicability of SEMT. Although the SEMT highlights the influence of the climate of respect on thriving at work, it does not differentiate between various types of respectful climates or examine the relationship between them. Respect can be viewed as the appropriate response and treatment given to others from a certain perspective. Negative workplace gossip breaches the norms of interpersonal communication by disseminating false information and harming individuals’ reputations. For those targeted by gossip, this behavior undeniably represents a disrespectful and improper form of interpersonal treatment. When organizational members initiate this type of disrespectful behavior, it can lead employees to perceive that the organization has breached and disrespected the mutual contract to create a fair and respectful work environment. In other words, the disrespect shown toward an individual by organization members can elicit a perception of not being respected by the organization. Consequently, negative workplace gossip can be interpreted as an act of disrespect by organizational members toward individuals, while a psychological contract breach can be viewed as an individual’s subjective perception of the organization’s disrespect toward them. This study classifies respectful climates from both member and organizational levels, and investigates how different types of respectful climates relate to one another and impact thriving at work, thus providing an integrated framework to understand the link between negative workplace gossip and thriving at work. The climate of respect also includes other elements, and future research could continue to explore the impact of other types of respectful climates on thriving at work.

Third, this study first reveals that *bianzhi* can moderate the effect of psychological contract breach on thriving at work, demonstrating that supportive factors in the work context may not always have positive effects. In the Chinese education system, *bianzhi* represents identity and can promote kindergarten teachers’ organizational identification (Liu et al., 2021). However, the process relating to social identity processes does not indicate that only favorable results can be anticipated. Highly identified employees often have higher expectations for the organization and its participants (Conroy et al., 2017). They may perceive more psychological contract breaches when the organization violates its promises or obligations to its workers. Therefore, teachers with *bianzhi* may be more negatively impacted by psychological contract breach. Previous studies have shown that work resources such as organizational tenure and supervisor support can buffer the adverse effects of negative factors on employees’ work attitudes and behaviors (Bradley, 2007; García-Cabrera et al., 2018). However, work resources do not always produce positive effects (Conroy et al., 2017; Li et al., 2022), which has been further confirmed by the results of this study. Furthermore, this study further expands the research on *bianzhi*. Prior studies mainly emphasized the positive effects of *bainzhi* on kindergarten teachers (He and Wei, 2022; Wang et al., 2022), neglecting potential problems that may arise from *bianzhi*. The present study suggests that organizations should focus on the context and dialectically consider the positive and negative effects of *bianzhi*. In addition, this study enriches the findings on the moderators of psychological contract breach. Previous studies mainly explored the boundary conditions of psychological contract breach in terms of subjective factors such as cognition (i.e., mindfulness) and personality traits (i.e., proactive personality) (Kayani and Alasan, 2021; Shaffakat et al., 2022). In the present study, the effect of *bianzhi* on psychological contract breach is firstly explored in terms of objective factors, which enriches the boundary research on the effect of psychological contract breach in the Chinese context.

### Practical implications

First, our findings indicate that organizations should implement effective measures to address negative workplace gossip regarding its impact on psychological contract breach and thriving at work. Awareness of negative workplace gossip should be enhanced, and transparent public policies should be implemented to achieve zero tolerance for negative workplace gossip. Moreover, clear procedures and protocols should be issued for reporting negative workplace gossip. In the Chinese cultural context with high power distance, some teachers may be reluctant to openly report negative workplace gossip to kindergartens. Regarding this problem, organizations can adopt anonymous reporting solutions, such as hotlines and mobile applications. Team-building activities should also be regularly organized to enhance mutual understanding and trust among teachers.

Second, our findings demonstrate the significance of alleviating psychological contract breach. Administrators can reduce the negative consequences of psychological contract breach by paying close attention to teachers’ emotional states and taking prompt measures to alleviate their negative emotions. For instance, kindergartens can provide counseling services for teachers to address negative emotions such as stress, anger, and depression (Zhao et al., 2007). In addition, administrators should increase their interaction and communication with teachers to understand their implicit expectations, thus preventing psychological contract breach and subsequent negative consequences.

Third, our empirical findings also indicate that more attention should be paid to teachers with *bianzhi* to minimize the aftereffects of negative workplace gossip and promote thriving at work. Due to the negative working environment, teachers may inevitably experience a breach of the psychological contract that undermines their thriving at work. The negative impact of psychological contract breach on teachers with *bianzhi* is even greater. Thus, kindergartens should dialectically consider the positive and negative effects of *bianzhi* in their management process. Special attention should be paid to the psychological contract of teachers with *bianzhi*. When kindergartens cannot fulfill their commitments or obligations due to objective factors, administrators should promptly and sincerely explain to teachers with *bianzhi*, thereby reducing the adverse impact of psychological contract breach on work behaviors.

### Limitations

Despite the theoretical and practical implications, this study has a few limitations. First, the convenience sample used in this study could cause sampling bias and reduce the validity of the results. Future research can make use of random sampling. Second, the self-report method in this study may result in bias, despite adopting reverse scoring. The results of this study can be further validated with more objective and unbiased data. Third, this study used cross-sectional data, making it necessary to interpret causality carefully. To illustrate the direction between negative workplace gossip, psychological contract breach, and thriving at work, longitudinal designs should be adopted in future research.

## Conclusion

In summary, this study deepens the understanding of how negative workplace gossip affects thriving at work among kindergarten teachers. By developing a moderated mediation model, the influencing mechanism is explored in detail. Specifically, negative workplace gossip is a risk factor for decreased thriving at work. Furthermore, negative workplace gossip affects thriving at work through the mediation of psychological contract breach, and *bianzhi* moderates the mediating model.

## Data availability statement

The original contributions presented in the study are included in the article/Supplementary material, further inquiries can be directed to the corresponding author.

## Ethics statement

The studies involving human participants were reviewed and approved by Ethics Committee for Scientific Research of Hubei Normal University. The patients/participants provided their written informed consent to participate in this study.

## Author contributions

CH proposed hypotheses, collected data, and completed paper writing. TF, JX, and HW analyzed data. All authors contributed to the article and approved the submitted version.

## Funding

This work was funded by Philosophy and Social Science Research Project of Hubei Provincial Department of Education (Grant No. 22Q121).

## Conflict of interest

The authors declare that the research was conducted in the absence of any commercial or financial relationships that could be construed as a potential conflict of interest.

## Publisher’s note

All claims expressed in this article are solely those of the authors and do not necessarily represent those of their affiliated organizations, or those of the publisher, the editors and the reviewers. Any product that may be evaluated in this article, or claim that may be made by its manufacturer, is not guaranteed or endorsed by the publisher.

## Appendix

Negative Workplace Gossip

1. As recently as one month ago, others (e.g., coworkers and/or supervisors) communicated damaging information about me in the workplace.

2. As recently as one month ago, others (e.g., coworkers and/or supervisors) spread unfavorable gossip about me in the workplace.

3. As recently as one month ago, others (e.g., coworkers and/or supervisors) made negative allegations about me in the workplace.

Psychological Contract Breach

1. Almost all the promises made by my employer during recruitment have been kept so far (reversed).

2. I feel that my employer has come through in fulling the promises made to me when I was hired (reversed).

3. So far my employer has done an excellent job of fulling its promises to me (reversed).

4. I have not received everything promised to me in exchange for my contributions.

5. My employer has broken many of its promises to me even though I’ve upheld my side of the deal.

Thriving at Work

1. I find myself learning often.

2. I continue to learn more and more as time goes by.

3. I see myself continually improving.

4. I am not learning (reversed).

5. I have developed a lot as a person.

6. I feel alive and vital.

7. I have energy and spirit.

8. I do not feel very energetic (reversed).

9. I feel alert and awake.

10. I am looking forward to each new day.
